# The Changing Climate and Pregnancy Health

**DOI:** 10.1007/s40572-022-00345-9

**Published:** 2022-02-22

**Authors:** Sandie Ha

**Affiliations:** Department of Public Health, School of Social Sciences, Humanities and Arts, Health Science Research Institute, University of California, Merced, 5200 N Lake Rd, Merced, CA 95343 USA

**Keywords:** Climate change, Environmental disaster, Extreme weather, Pregnancy health, Perinatal health

## Abstract

**Purpose of Review:**

Climate change is the biggest public health threat of the twenty-first century but its impact on the perinatal period has only recently received attention. This review summarizes recent literature regarding the impacts of climate change and related environmental disasters on pregnancy health and provides recommendations to inform future adaptation and mitigation efforts.

**Recent Findings:**

Accumulating evidence suggests that the changing climate affects pregnancy health directly via discrete environmental disasters (i.e., wildfire, extreme heat, hurricane, flood, and drought), and indirectly through changes in the natural and social environment. Although studies vary greatly in design, analytic methods, and assessment strategies, they generally converge to suggest that climate-related disasters are associated with increased risk of gestational complication, pregnancy loss, restricted fetal growth, low birthweight, preterm birth, and selected delivery/newborn complications. Window(s) of exposure with the highest sensitivity are not clear, but both acute and chronic exposures appear important. Furthermore, socioeconomically disadvantaged populations may be more vulnerable.

**Summary:**

Policy, clinical, and research strategies for adaptation and mitigation should be continued, strengthened, and expanded with cross-disciplinary efforts. Top priorities should include (a) reinforcing and expanding policies to further reduce emission, (b) increasing awareness and education resources for healthcare providers and the public, (c) facilitating access to quality population-based data in low-resource areas, and (d) research efforts to better understand mechanisms of effects, identify susceptible populations and windows of exposure, explore interactive impacts of multiple exposures, and develop novel methods to better quantify pregnancy health impacts.

## Introduction

Climate change is the long-term change in the average weather patterns that define local, regional, and global climates. In 2007, the Intergovernmental Panel on Climate Change (IPCC) presented a large body of evidence supporting the presence of global warming and the impact of anthropogenic activities on the global climate [1]. The report suggests that a child born today will be living in an environment that is more than four degrees warmer than the average temperature during the preindustrial period and will experience significantly more frequent and intense environmental disasters such as heatwaves, wildfires, and hurricanes [2]. Since then, the number of published articles on health impacts of climate change increased by 182% [3]. These studies suggest that climate change is associated with many short- and long-term health effects across the human lifespan, ranging from dehydration to heatstroke, respiratory diseases, infectious diseases, mental health complications, cardiovascular disease, and even death [2–4]. As such, climate change is recognized as the “biggest global health threat of the twenty-first century.” [5].

Pregnant women and the growing fetus experience an extraordinary time with many tightly regulated physiologic and psychologic changes [6, 7]. Any environmental perturbation during this sensitive period could have both immediate and life-long consequences for both mother and offspring [8, 9]. However, research on the health impacts of climate change on pregnancy outcomes is highly limited [10], contributing to the lack of consistent guidelines on how to adapt to and/or mitigate climate impacts among pregnant women. In fact, pregnant women have only been recently added as a vulnerable group with respect to environmental exposures such as air pollution and extreme heat [11, 12]. The objective of this narrative review is to summarize recent literature regarding how climate change and related environmental disasters influence pregnancy health.

**Fig. 1:**
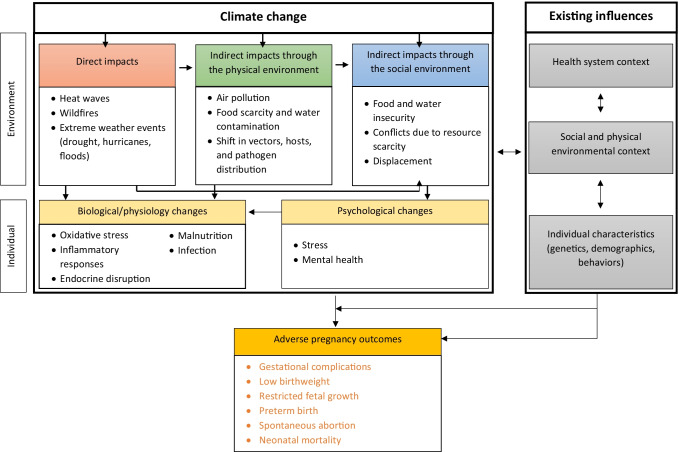
Impacts of climate change on pregnancy outcomes

Climate impacts on pregnancy health can be conceptualized to involve (a) direct impacts via discrete environmental disasters, (b) indirect impacts through changes in the natural environment, and (c) indirect impacts through changes in the social environment (Fig. 1). It is important to note that although direct and indirect impacts are commonly evaluated separately, they often occur simultaneously and have a synergistic and/or cascading impact on pregnancy health.

## Direct Impacts of Climate Change on Pregnancy Outcomes

Climate change is expected to cause more frequent and intense climate-related environmental disasters such as heatwaves; wildfires; and extreme weather events such as drought, hurricane, and flood [1, 13]. As described below, accumulating evidence suggests positive associations between prenatal exposures to these events and adverse pregnancy outcomes. More importantly, populations who have the least access to the world’s resources and contribute least to climate change bear a disproportionately higher burden. [5]

### Heat Events

The average annual number of heatwaves, defined as a series of unusually hot days, in the USA increased from two in the 1960s to six in the 2010s. [14] The length and average temperature of individual heatwaves have also increased significantly in recent decades [14]. Pregnant women are more prone to heat stress than non-pregnant women due to their compromised thermoregulation and homeostasis ability. A recent meta-analysis of 70 studies across 27 countries examines the impact of high temperature on preterm birth, low birthweight, and stillbirth [15]. Summary estimates suggest a 16% higher risk of preterm birth during heatwave days compared to on non-heatwave days [15]. Furthermore, each additional degree Fahrenheit (0.56 °C) was associated with a 5% increased risk. Potential susceptible exposure windows for preterm birth include 1 month preconception, the month of conception, first trimester, second trimester, and last week of pregnancy, suggesting both acute and chronic exposures are relevant [15]. The meta-analysis also showed that the low birthweight rate was 9% higher during periods with hotter than usual temperature, with babies on average 26 g lighter [15]. Additionally, stillbirth risk was 46% higher during heatwave compared to non-heatwave days, with risk increment of 5% for each additional degree Fahrenheit. Early pregnancy appears to be the most susceptible window of exposure for stillbirth [15, 16]. Meanwhile, high temperatures have also been linked to other serious pregnancy outcomes such as premature rupture of membranes [17], gestational cardiovascular events [18], gestational hypertension and preeclampsia [19, 20], birth defects [21], and neonatal mortality. [22].

Generally, both acute and chronic exposures to heat appear to have an impact on pregnancy health, but critical periods of maternal sensitivity have not been clearly identified. [15, 23–27] Acute exposures are often evaluated as the daily temperature during the week prior to the event (i.e., preterm delivery, stillbirth), and chronic windows are mostly assessed as average temperature across specific trimesters and/or the entire gestation [15, 25, 26]. Subgroups with heightened vulnerability to heat impacts are not clear but may include those with extreme child-bearing ages, lower socioeconomic positions, lower educational attainment, or come from minoritized populations [15]. On the other hand, emerging evidence suggests that greenspace can help mitigate the impact extreme temperatures on pregnancy outcomes. [28].

The exact mechanisms linking ambient temperature to adverse pregnancy outcomes are not fully understood but likely involve a few inter-related pathways. Prolonged heat exposure leads to heat stress and triggers inflammatory and oxidative stress responses that promote endothelial dysfunctions and increase blood viscosity [29–32]. These effects, coupled with increased peripheral blood flow for heat dissipation, lead to decreased uterine blood flow and impaired oxygen and nutrients transfusion to the developing fetus [20, 33–36]. Heat exposure can also stimulate maternal antidiuretic hormone and oxytocin, both of which reduce uterine blood flow, and switch fetal metabolism from anabolic to catabolic pathways [37]. Extremely low or high temperatures are also associated with emotional stress during pregnancy, which may further exacerbate endocrine, endothelial, and placental dysfunctions. [38].

### Wildfires

The number of wildfires and area burned increased significantly in recent years and is expected to continue [14]. A recent meta-analysis reported that, as of June 2020, eight epidemiologic studies in four countries (covering a total of ~ 1.7 million births) have evaluated the effects of wildfire exposures on adverse pregnancy outcomes [39]. The analysis shows birthweight is most consistently impacted by wildfires. More specifically, six of the seven studies that evaluated associations between wildfire exposures and birthweight reported a significant link with birthweight reduction [39]. Two studies (out of four) identified associations between wildfire exposures with preterm birth risk [40, 41], and one study with fetal loss and infant mortality [42]. A more recent California study suggests that each additional day of exposure to wildfire smoke, as estimated by a satellite-based approach, was associated with ~ 0.5% increase in the risk of preterm birth [43]. The authors also estimated that wildfires may contribute to almost 4% of observed preterm births. Few studies evaluated critical windows of exposure, although those that did suggest that the second and third trimester may be more sensitive, and those from poorer neighborhoods and smokers may be more vulnerable [27, 39, 40, 43, 44]. Of note, there is significant heterogeneity between studies, especially in how exposure is defined, which include distance to wildfires, fine particle concentration, heat spots from satellite images, and aerosol index. Some studies compared exposures between areas with varying degree of exposures, while others compared exposures between time periods with and without wildfires.

Although research on the impacts of wildfires on pregnancy health is still in its infancy, much more mature literature on the effects of combustion products, smoking, and air pollution on pregnancy health can potentially inform what we can expect from wildfires. Wildfire smoke is a complex mixture containing gaseous pollutants, organic compounds, and fine particles, much of which is similar to pollution from combustion, smoking, and other sources [45, 46]. These pollutants may impact pregnancy health through a few mechanisms including endothelial dysfunction, endocrine disruption, immunologic dysfunction, systemic inflammation, and oxidative stress [47–51]. These changes ultimately reduce maternal-placental-fetal blood flow and nutrient/oxygen exchange or induce epigenetic changes that can impact fetal growth [52]. Pollutants can also influence fetal outcomes through induction of genetic, epigenetic, or morphological changes in paternal germ cells [53–55]. Given the large literature suggesting consistent link between air pollution and adverse pregnancy outcomes including stillbirth, birth defects, restricted fetal growth, preterm birth, and selected maternal/fetal complications, wildfires are expected to have similar impacts and warrant further investigation. [56–60].

Wildfires can also cause psychological distress due to financial and personal loss, as well as disruption of infrastructure such as housing, healthcare, work, communication, and transportation [61]. People exposed to wildfires report higher levels of distress and more symptoms of somatization, depression, anxiety, hostility, phobic anxiety, and psychopathology [62–64]. They also have increased risk of post-traumatic distress disorder (PTSD), and insomnia [65, 66], persisting even 1 year later [67]. These mental health complications are known to increase the risk of pregnancy outcomes including gestational complications, pregnancy loss, preterm delivery, and low birthweight [68–70]. During wildfire events, psychological stressors may have synergistic impact with the biological mechanisms discussed above to amplify the risk of adverse pregnancy outcomes.

### Extreme Weather Events

The frequency and intensity of extreme weather events such as hurricanes and floods are expected to increase. Hurricanes reduce access to safe food and water, and induce stress by disruption of existing infrastructures [71–74]. During a hurricane, pregnant women report significant fear of losing home and/or job, and serious concerns about the health of their baby and the birth process [75–77]. Those with severe hurricane experience also report higher frequency of mental health complications such as PTSD and depression, which are all major risk factors for subsequent adverse perinatal outcomes [78]. According to a recent review, as of February 2020, 19 studies evaluated hurricane exposures in relation to pregnancy outcomes [79]. There is a high degree of heterogeneity in how hurricane exposures were defined and assessed. Some exposure assessment strategies include distance from storm path, properly damage, residence in nationally designated disaster areas, maximum wind speed, or questionnaires that include specific impact scales. Pregnancy outcomes generally include gestational age, birthweight, gestational complications, fetal death, and/or obstetric and neonatal complications. Regardless of the approach, studies suggest that women with prenatal hurricane experience have greater risk of hypertensive disorders of pregnancy, labor and birth complications, C-section, and newborn complications [79]. Increased risk of preterm birth and low birthweight is also reported, but these findings are less consistent in part due to high heterogeneity between studies.

More recently, Xiao et al. 2021 reported that Hurricane Sandy (New York, 2012) was associated with a 4.1% increased rate of emergency department (ED) visits within 1 week for eight major pregnancy complications [80]. The authors also found that the rate of ED visits for pregnancy complications increased by 16.6% within 7 days after power outage. Duration and severity of power outage were also related to risk. Similarly, Pan et al. 2021 reported that exposure to Hurricane Michael (Florida, 2018) was associated with a 39% increased risk of delivering a small for gestational age baby, and a 19% increased risk of having inadequate prenatal care, suggesting that disruption to prenatal care may be a pathway to pregnancy risk [72]. The risks were also more pronounced in the most affected areas defined by the Federal Emergency Management Agency’s disaster declarations.

Floods are the most common natural disaster globally [81]. They can affect pregnancy health by disrupting infrastructure, limiting access to safe food and water, facilitating the spread of waterborne pathogen and certain vectors, and creating the opportunity for unintentional distribution hazardous chemicals such as heavy metals and toxic pesticide compounds [82–85]. Health impacts of floods are commonly evaluated together with hurricanes as they often occur simultaneously. Thus, their independent effects on adverse pregnancy outcomes are difficult to evaluate. Nevertheless, evidence suggests that their perinatal impacts range from reduced access to a quality diet during pregnancy to maternal stress, pregnancy complications, and even maternal and perinatal mortality. [81].

The flood caused by Hurricane Harvey (Texas, 2017) was associated with a 24% higher rate of ED visits for pregnancy complications, and the risk remained elevated 1 month after the event [86]. Hurricane Katrina’s flood (Gulf Coast US, 2005) was associated with a 23% increased rate of preterm birth in Alabama [87] and 3% higher rate of low birthweight in Louisiana [88]. Similarly, Hurricane Katrina’s and Rita’s combined floods (Louisiana, 2005) were associated with 40% increased rates of fetal death in areas with damage to 10–50% of housing, and 140% in areas with more than 50% damage, when compared to areas with < 10% damage [89]. Additionally, each percent increase in destruction of housing stock caused by these floods was associated with 1.7% higher rates of fetal death.

Flooding associated with Hurricane Andrew (Florida, 1992) was associated with 20% higher risk of fetal stress [90], defined as presence of a deficiency in oxygen reaching fetal tissues, and with 20% higher rate of C-section [91] Similarly, the rates of low birthweight and preterm birth increased 11% and 9% after the Red River Flood (North Dakota, 1997) [92]. A prospective study of a subsequent Red River Flood (North Dakota, 2009) found that women who lived closer to the flood during the first trimester had significantly smaller babies (i.e., − 43 g per mile) [93]. Similarly, women who were displaced by the 2011 Thailand flood had babies on average 175 g lower compared to unaffected women [94]. Meanwhile, older flood events such as those from Hurricane Agnes (New York, 1972) [95] and the Great Flood of 1997 in Poland [96] were documented as leading to increased rates of spontaneous abortion. On the other hand, a difference-in-difference analysis of the 2013 Calgary flood compared adverse pregnancy outcomes in flooded and non-flooded areas and in affected and unaffected time periods suggests no associations with preterm birth, small for gestational age, or preeclampsia; but a small increase in the incidence of gestational hypertension. [97].

The predicted trend of drought occurrence is less consistent [98], but recent evidence suggests that frequency, duration, and intensity have increased in parts of the Americas, Africa, and Asia, and this trend is expected to continue [99, 100]. Like hurricanes, droughts lead to water and food insecurity and malnutrition due to reduced access to safe drinking water, massive livestock death, and crop failure [101, 102]. In most sub-Saharan African countries where droughts are endemic, more than 20% of women are classified as malnourished, a maternal major risk factors for low birthweight and fetal growth restriction [103]. Studies on the direct impact of droughts on pregnancy in humans are limited because severe droughts often occur in areas with limited resources, data, and research infrastructure. Nevertheless, a study in Zambia shows that during the Southern African drought of 2001–2002, food prices increased, leading to unfavorable maternal micronutrient status and decreased infant length [104]. Extended drought may also lead to famine, exposure to which is widely recognized as a significant cause of adverse pregnancy outcomes, subsequent chronic diseases later in life, and even intergenerational health impacts. [105–109].

### Rising Sea Level

The sea level has risen 6–8 in (15–20 cm) in some areas since 1993 [110]. This increase is mostly due to melting of glaciers and ice sheets, and thermal expansion of water as it warms. Approximately 40% of the world’s population lives in coastal areas and may be vulnerable to rising sea level as storms and hurricanes can push further inland and affect a greater proportion of the population [110]. This also means more frequent tide-associated flooding, causing additional risk of displacement, infrastructural instability, and exposures to mold and other potentially hazardous chemicals for pregnant women.

## Indirect Impacts Through the Natural Environment

### Air Quality

The changing climate has direct impact on both outdoor and indoor air quality. Wildfires release large amounts of carbon dioxide, black carbon, ozone precursors, volatile organic compounds including polycyclic aromatic hydrocarbons, and many other hazardous air pollutants. In addition, meteorological conditions such as extreme temperatures can facilitate the formation of pollutants such as ozone and fine particles. In certain areas, warmer temperatures may also enhance exposure due the increased likelihood of outdoor activities and infiltration of outdoor air into homes as a result of people leaving doors and windows open [111, 112]. Unless effectively mitigated by emission reduction strategies, air pollution levels will increase as environmental conditions become more conducive for air pollution emission and formation [113]. Studies around the world, regardless of design, consistently suggest that preconception and prenatal exposures to ubiquitous gaseous pollutants and fine particles increase the risk of adverse pregnancy outcomes including gestational hypertension, gestational diabetes, pregnancy loss, preterm birth, and restricted fetal growth [56, 59, 114–117]. The biologic mechanisms linking air pollution and adverse pregnancy outcomes are briefly discussed in the “Wildfires” section. Meanwhile, a changing climate can also influence the production, distribution, and seasonality of aeroallergens such as pollen. Studies show that warmer temperatures in the USA have caused more than 21% higher pollen concentration together with an increase of more than 20 days in the pollen season from 1990 to 2018 [118]. A few studies reported associations between prenatal exposures to pollen and subsequent health risks for the offspring including asthma hospitalization within the first year of life and atopic diseases during childhood [119, 120]. In a large cohort of more than 225,000 singleton births, Kavigne et al. 2017 demonstrated that daily counts of pollen and fungal spores are positively associated with preterm birth rates. [121].

### Food and Water Quality and Availability

Extreme climate-related disasters decrease crop productivity, kill livestock, increase food spoilage, and slow food supply distribution, all of which can lead to food insecurity and malnutrition. In many parts of the world, food scarcity is already a significant public health problem, especially for pregnant women from lower socioeconomic positions. Food insecurity has been found to be associated with the risk of major birth defects (e.g., cleft palate, dextro-transposition of the great arteries, tetralogy of Fallot, spina bifida, and anencephaly) [122], low birthweight, preterm labor, gestational diabetes, and gestational hypertension [123–128]. In areas without proper refrigeration, extreme weather events can increase opportunity for food spoilage, leading to risk of food-borne illness in pregnant women and ultimately adverse pregnancy complications [113, 129–131]. The change in insect and fungal distribution may lead to increase pesticide use, resulting in increased exposures to pesticide drift and higher concentrations of pesticides in food [132, 133]. Pesticide exposures through maternal intake or residential proximity to agricultural pesticide application sites (e.g., farms) have been consistently shown to increase fetal health risk such as birth defects and subsequent neurodevelopmental complications. [134–142].

Currently, as much as one billion people worldwide do not have access to safe water [143]. With global water consumption increasing rate twice as fast as population growth, water shortages will likely threaten pregnancy health, especially in regions with fewer resources [144]. Water has implications not only for drinking but also for sanitation, a major contributor to the global burden of disease [145, 146]. Although the number of studies on water availability and pregnancy outcomes is still limited, existing evidence suggests that limited water access can cause dehydration among pregnant women, leading to increased risk low birthweight and preterm birth [147–150]. Lack of safe water supply can also increase the likelihood of consumption of contaminated water resulting in poor pregnancy health outcomes. [151–153].

### Shifts in Vector and Pathogen Distribution

The World Health Organization reports that in 2017, 435 million of the global population rely on water from unprotected sources such as wells and springs, and 144 million depend on untreated surfaces such as lakes, ponds, rivers, and streams [154]. Water shortages, coupled with warming temperature and extreme weather events, lead to even more opportunities for widespread distribution of and exposures to common water-borne pathogens such as cyanobacteria, enteric bacteria, parasites, and *Vibrio* bacteria [155]. Pregnant women can be exposed to these pathogens through drinking, recreational use, and/or ingestion of shellfish [156]. Waterborne infections are known to cause many pregnancy and fetal complications including maternal biliary ascariasis, septicemia in pregnancy, spontaneous miscarriage, preterm delivery, intrauterine growth restriction, and birth defects. [157].

The distribution and activity of some important vectors such as ticks and mosquitoes have expanded northward and to higher elevations in recent years. Accordingly, the incidence of malaria is expected to increase in previously unaffected areas, which will be further exacerbated by increased population density and forced migration caused by extreme weather events and food/water shortage [158]. Malaria infection has been known to cause severe malaria-induced anemia during pregnancy and increase the risk of intrauterine growth restriction, preterm birth, and low birthweight [159, 160]. Meanwhile, dengue transmission is also expected to increase, with approximately 5–6 billion people considered at risk by the end of the century [143]. Maternal dengue infection is capable of vertical transmission to the fetus, causing fetal or perinatal mortality. This infection is also known to increase the risk of maternal mortality, pre-eclampsia, eclampsia, preterm birth, low birthweight, and C-section  [161, 162].

## Indirect Impacts Through the Social Environment

Frequent extreme weather events and food and water insecurity are already causing displacement and forced migration globally. By 2050, the world expects to have several hundreds of millions of climate refugees as a result of droughts, natural disasters, rising sea levels, and lack of food and water [5]. Even for those who do not evacuate, significant loss in infrastructure such as housing, work, and healthcare can be traumatizing. Globally, women bear a disproportionately higher burden of family responsibilities during and after crises compared to men. This, coupled with gendered disparities in financial and economic instability, makes women particularly vulnerable to the social impact of climate change [163, 164]. It is estimated that women account for more than 75% of the displaced population [165]. As food, water, and shelter become survival priority, pregnant women are less likely to seek prenatal care. In addition, high-risk pregnancies involving gestational complications (e.g., gestational diabetes, pre-eclampsia) may not be diagnosed, resulting in poor perinatal outcomes.

The high degree of forced migration may create tension between groups, leading to increased frequency of social conflicts [166]. There is a very limited understanding of the impacts of such social threats on pregnancy health, but the stress associated with social unrest is traumatizing and likely affects pregnancy outcomes negatively [167, 168]. Studies also show that violence against women increases in times of crises while escaping pathways are limited. [163].

Even in areas not impacted by forced migration, rising temperatures in cities densely populated areas can form urban heat islands, which are areas that are significantly warmer than their surroundings as a result of land use and waste heat generated by energy use. Many US cities have temperatures up to 7°F (~ 4°Celsius) warmer than their surroundings [169]. Although the impact of heat island on pregnancy health has not received attention, studies have shown that minoritized populations are more likely to be exposed, which may further contribute to reproductive health disparities. [170, 171].

## Implications

**Fig. 2:**
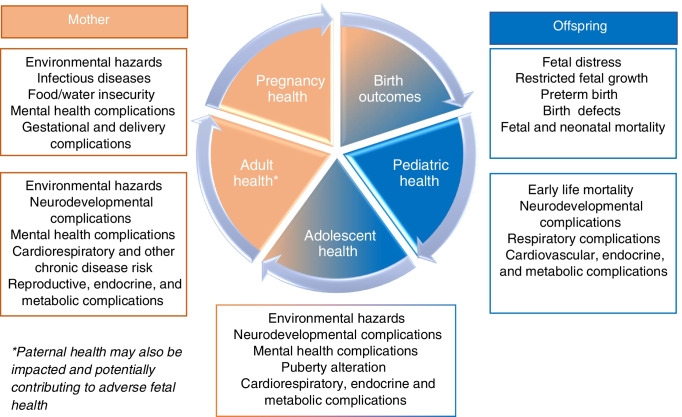
The cycle of health impacts following climate related exposures during pregnancy

An increasing body of evidence suggests that climate change adversely impacts pregnancy health through both direct and indirect pathways. The Developmental Origin of Health and Disease theory [8] suggests environmental perturbation(s) during pregnancy (and other developmentally critical periods) have significant immediate and long-term health impact for both mother and offspring (Fig. 2). Pregnant women who have pregnancy complications are more likely to experience recurrence in subsequent pregnancy [172] and have greater risk of cardiovascular and metabolic diseases later in life [173, 174]. Babies affected by preterm birth and low birthweight are more likely to develop subsequent health complications including neurodevelopmental disorders, immunologic complications, obesity, and cardiovascular diseases, all of which put them at higher risk of adverse pregnancy outcomes if they become pregnant [175]. Accordingly, the impact of climate change on pregnancy health is not limited to this time window, but may propagate health risk across an individual’s lifespan and even into future generations (Fig. 2). Although beyond the scope of this review, the health impacts of the changing climate on paternal health can also further contribute to adverse fetal outcomes. [176].

Despite challenges, concerted efforts for adaptation and mitigation should be continued and strengthened. First, educational efforts and resources to raise awareness will facilitate behavioral changes and encourage public support for actions needed to reduce emissions and mitigate health impacts. Although climate change is generally known to the public, populations most impacted by its consequences have limited knowledge, power, and resources to mitigate its impact [177]. Thus, it is pertinent that any adaptations and mitigation efforts target these underserved populations. Such efforts should also target healthcare professionals, who, through patient-provider relationship, can support vulnerability reduction strategies. A US survey of health professional suggests that nearly 0% of OBGYN practitioners discuss environmental impacts of health with their patients [178]. Data also show that the majority of healthcare providers recognize the presence of climate change as a major threat to human health [179] but the lack of time, training, resources, and guidance are major barriers. Thus, provision of training, patient educational materials, and clear policy guidance will empower healthcare providers to become a critical part in health mitigation efforts.

Meanwhile, local, regional, and international policies aiming to reduce emission should be continued and strengthened. Even with massive international agreements such as the Kyoto Protocol and Paris Agreement, some experts are concerned that they are not aggressive enough. Solutions could include strengthening the commitment and implementation of strategies for accountability. Local policies have also proven to be extremely important. When the Trump administration withdrew from the Paris Agreement, more than 600 local governments still had detailed climate action plans [180]. Furthermore, efforts to reduce waste and increase renewable energy consumption, energy efficient appliances, and carbon-neutrality should be reinforced.

Lastly, more research is needed as we are only at the beginning of understanding how climate change and its environmental consequences impact pregnancy health. Research efforts are especially needed in areas with the most impact, which are often underserved with few resources. Research priorities should include the following:

1. Development of novel methods and models to better quantify climate change and its impacts on health, while considering confounding and the complex interaction between extreme climatic events, and between individual and environmental factors
2. Identification of susceptible window(s) of exposure and sensitive subgroups for specific climatic events
3. Further understanding of the biological and social mechanisms linking climate change and adverse pregnancy outcomes.
4. Leveraging multidisciplinary and multilevel collaboration to identify and evaluate strategies to adapt to or mitigate health impacts for pregnant women
5. Facilitation of availability, accessibility, and timeliness of population-based environmental and health data, especially in hard-hit areas
6. Consensus on important study design aspects and reporting strategies that can support decision making at local, regional, national, and international level

## Conclusions

Climate change is considered the biggest public health threat of the twenty-first century. Pregnant women and the growing fetus, especially those from areas with less resources, are particularly vulnerable to its direct and indirect impacts. Policy, clinical, and research strategies to adapt to or mitigate the effects of climate change are now more important than ever. A successful solution should involve close collaboration of interdisciplinary and multilevel bodies including the government, community partners, the public, physicians, industry partners, public health practitioners, and researchers.

## Data Availability

Not applicable.
